# TCDD-Induced Activation of Aryl Hydrocarbon Receptor Inhibits Th17 Polarization and Regulates Non-Eosinophilic Airway Inflammation in Asthma

**DOI:** 10.1371/journal.pone.0150551

**Published:** 2016-03-03

**Authors:** Xiao-ming Li, Juan Peng, Wen Gu, Xue-jun Guo

**Affiliations:** Department of Respiratory Medicine, Xinhua Hospital, Shanghai Jiao Tong, University School of Medicine, Shanghai 200092, China; McGill University, CANADA

## Abstract

The aryl hydrocarbon receptor (AhR), a transcription factor of the bHLH/PAS family, has recently been demonstrated to regulate T cell differentiation. Whether AhR activation participates in allergic airway inflammation remains unknown. In the current study, using a non-eosinophilic asthma model, we demonstrate that 2, 3, 7, 8-tetrachlorodibenzo-P-dioxin (TCDD), a potent AhR ligand, reduced the airway infiltration of neutrophils, airway hyperresponsiveness and Th17 cytokine expression. Furthermore, stimulation with TCDD promoted Treg differentiation and inhibited Th17 differentiation. However, the maturation of dendritic cells may not be inhibited by AhR activation. This study thus indicates a critical role of TCDD-induced AhR activation in the regulation of non-eosinophilic airway inflammation.

## Introduction

Asthma, a complex respiratory disease, is characterized by airway inflammation, bronchial hyperresponsiveness and airway structural remodeling [1, 2]. It is driven by Th2 and Th17 cell differentiation and activation, which leads to eosinophilic and neutrophilic airway infiltration, respectively. Recent studies have demonstrated that neutrophilic airway inflammation primarily occurs in more severe asthma [3, 4, 5]. Dendritic cells play a key role in T cell differentiation and result in various Th subsets [6]. The functional impairment of regulatory T lymphocytes (Tregs) boosted the activation of effector T lymphocytes via the production of proinflammatory dendritic cells [7, 8]. Therefore, the effective regulation of T cell differentiation has important value in controlling airway inflammation in asthma.

The aryl hydrocarbon receptor (AhR) is a ligand-activated transcription factor that belongs to the bHLH-PAS protein family [9, 10]. All major cell types, including bronchial epithelial cells, express AhR [11]. AhR activation leads to receptor translocation from the cytosol to the nucleus and subsequent binding to its dimerization partner, the aryl hydrocarbon receptor nuclear translocator (ARNT). The AhR-ARNT complex binds to an enhancer sequence of drug-metabolizing enzymes, such as cytochrome P450 1A1 (CYP1A1), which is important for detoxification [12]. AhR activation has recently been demonstrated to play an important role in immune system regulation [13, 14]. Moreover, several studies have indicated that AhR activation regulates the differentiation of both Th17 and Tregs [15, 16]. Quintana *et al* reported that AhR activation by its ligand 2, 3, 7, 8-tetrachlorodibenzo-p-dioxin (TCDD) induced functional Tregs and suppressed experimental autoimmune encephalomyelitis. Moreover, AhR activation by 6-formylindolo [3, 2-b] carbazole (FICZ) enhanced Th17 differentiation and aggravated experimental autoimmune encephalomyelitis in mice [17]. Thus, we hypothesized that TCDD-induced activation of AhR may play an immunoregulatory role in airway inflammation in asthma.

The current findings demonstrated that TCDD alleviated airway inflammation by decreasing neutrophil recruitment and Th17 cytokine expression. Moreover, stimulation with TCDD promoted Treg differentiation and inhibited Th17 differentiation. Furthermore, TCDD-induced activation of AhR enhanced FOXP3 expression but reduced RORγ expression. However, the maturation of dendritic cells may not be inhibited by AhR activation. Taken together, our findings indicate a critical role of TCDD-induced AhR activation in the regulation of non-eosinophilic airway inflammation.

## Materials and Methods

### Mice

Six week-old female BALB/c mice were purchased from Shanghai SLAC Co. (Shanghai, China). All mice were maintained under pathogen-free conditions at the animal center of Xinhua Hospital. The health of the mice was monitored every other day. All mice were divided into 3–4 groups, and each group comprised 5 mice. All animal care and handling protocols were approved by the Institutional Animal Care and Use Committee at Xinhua Hospital (Shanghai, China). All surgery was performed under sodium pentobarbital anesthesia, and all efforts were made to minimize suffering.

### Induction of non-eosinophilic asthma and TCDD treatment

**Fig 1:**
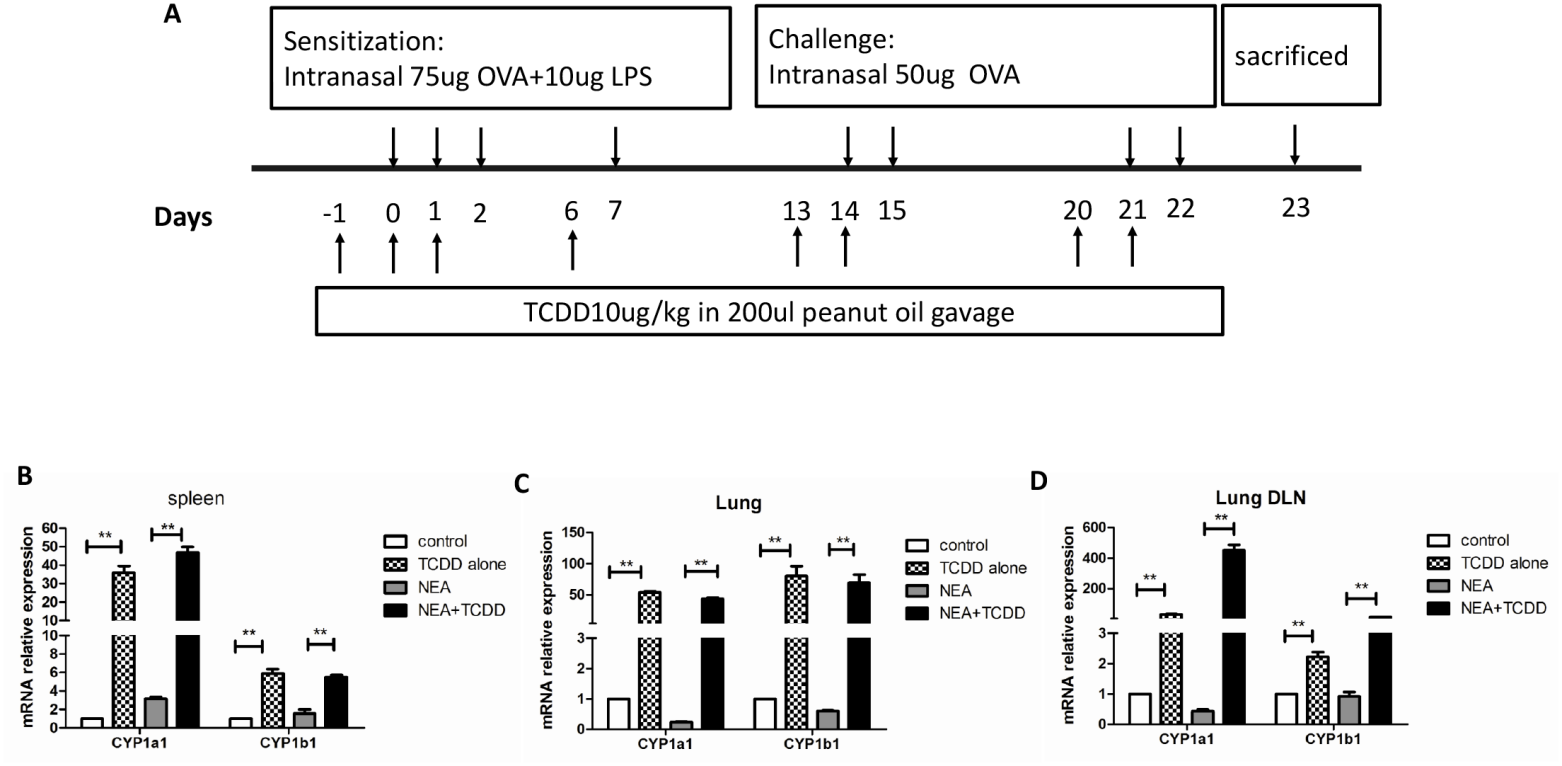
AhR activation by TCDD promoted the expression of CYP1a1 and CYP1b1 genes. Mice were intranasally sensitized with OVA and LPS, and TCDD was gavaged one day prior to being sensitized and challenged. (A) Timeline of the OVA/LPS immunization/challenge and the TCDD treatment protocol. (B) mRNA expression of CYP1a1 and CYP1b1 in spleens was determined via real-time PCR. (C) mRNA expression of CYP1a1 and CYP1b1 in lungs was determined via real-time PCR. (D) mRNA expression of CYP1a1 and CYP1b1 in lung-draining mediastinal lymph nodes was determined via real-time PCR. N = 5 per group and *P<0.05, **P<0.01.

We generated a non-eosinophilic asthma(NEA)model, which indicates that the inflammatory cells in the airway are not mainly eosinophils, as described by Kim and his colleagues [18]. In brief, 6-wk-old mice were intranasally sensitized with OVA (75 μg) and LPS (10 μg) (Sigma-Aldrich) on days 0, 1, 2, and 7 and then challenged with OVA (50 μg) alone on days 14, 15, 21, and 22. The control group was simultaneously administered PBS. To determine the effect of TCDD on airway inflammation, the mice were gavaged (10 μg/kg) one day prior to being sensitized and challenged, which reflects a total of eight times (Fig 1A). TCDD (purity 99.1%, 50 μg/ml in toluene) was purchased from AccuStandard, Inc. and diluted in peanut oil. The control groups were simultaneously gavaged with peanut oil. Twenty-four h after the last challenge, the mice were euthanized, and the sera, cBALFs, lungs, spleens and lung-draining mediastinal lymph nodes were harvested and analyzed.

### Measurement of methacholine AHR

Twenty-four hours after the last challenge, measurements of the airway hyper responsiveness (AHR) were performed using a Buxco’s modular and invasive system (Buxco Electronics Inc., NY, USA). Changes in the airway resistance (RL) and lung dynamic compliance (Cdyn) were measured as described by Amdur and Mead [19]. Briefly, each anesthetized mouse was tracheostomized and intubated with an appropriate cannula and subsequently laid supine inside the body plethysmograph chamber connected to the ventilator. After a stable baseline airway pressure (<5% variation over 2.5 min) was achieved, the mice were administered aerosolized PBS or various concentrations of methacholine (3.125, 6.25, 12.5, or 25 mg/ml) via a jet nebulizer into the head chamber. The minimum values for the RL and Cdyn were calculated in response to increasing concentrations of inhaled methacholine; the values were expressed as a percent change from the baseline value as previously reported [20].

### Lung histology

The right lung was removed and immediately fixed in 4% buffered paraformaldehyde for 24 h at room temperature. Paraffin-embedded sections were created and stained with hematoxylin and eosin (H&E) or periodic acid-Schiff (PAS). The airway inflammation and mucus production were estimated using light microscopy under ×200 magnifications. Samples of the sections were incubated overnight at 4°C with rabbit-anti-MPO (Abcam) and rabbit-anti-Ly-6G (Gr-1) (Biolegend) antibody to identify the infiltration of neutrophils. The immunostained sections were quantitatively characterized via digital image analysis using Image Pro-Plus 6 software. The percentage of the positive staining area of the airway was counted from 10 randomly selected fields per section.

### Analysis of the sera and BALF

Blood was harvested via cardiac puncture. The serum was collected after 2–3 hours of coagulation and 15 minutes of centrifugation at the speed of 500 g. The tracheas of the mice were lavaged three times via a catheter with 0.8 ml sterile PBS. The BALF was centrifuged for 5 minutes at 300 g, and the supernatant was harvested. The precipitated cells were resuspended in 20 μl PBS for cellular composition using May-Gruenwald Giemsa staining. The supernatants from the sera and BALF were stored at -80°C until cytokine level analysis.

### Cell isolation and culture

The spleens and lung-draining mediastinal lymph nodes were removed from the mice. The tissues were grind in Dhanks balance buffer on a mesh. The suspension was collected and centrifuged at a speed of 500 g for 10 minutes. Erythrocytes were lysed using a red blood cell lysis buffer. The cells were resuspended in RPMI 1640 medium with 10% bovine serum and further cultured with OVA (0, 10, 30 and 100 μg/ml) for 3 days; the supernatants were analyzed for cytokine expression by ELISA (eBioscience).

### Cell staining and Flow cytometry

For intracellular cytokine analysis, the cells from the spleen and lung-draining mediastinal lymph node were restimulated with leukocyte activation cocktail (BD Bioscience) 2 μl/ml for 5 hours. The cells were permeabilized and stained with a Cytofix/Cytoperm kit (eBioscience). CD4-FITC, CD25-PE, and IL-17-PE-Eflour-610 (eBioscience) were used to label Treg and Th17 cells. The analysis was performed on a BD FACSCalibur (BD Bioscience).

### Real time PCR

Total RNA was extracted using TRIzol reagent (Invitrogen), and cDNA was synthesized using a PrimeScript TM RT reagent kit (Takara). The gene expression was examined with an ABI 7500 real time PCR system using a SYBR Premix Ex Taq TM II kit. The data were normalized to a reference, Actb. The following primer pairs were used: CYP1a1 forward, TTCCTGTCCTCCGTTACCTG, and reverse, GCCCTTCTCAAATGTCCTGT. CYP1b1 forward, GAGAGAGTGCCATC CACCAG, and reverse, GTAGTGACCGAACGCCAGAC. FOXP3 forward, GCCCATCCAATAAACTGTGG, and reverse, GTATCCGCTTTCTCCTGCTG. RORγ forward, CCTCCTGCCACCTTGAGTAT, and reverse, TCTGGACCCTGTTCTGGTT.

### Measurement of DC maturation in Lung mononuclear cells

Lung tissue was chopped and incubated in 37°C with 300 μg/ml collagenase (Sigma) and 42 μg/ml of DNase (Sigma). Following digestion for 90 min, lung mononuclear cells were attained using lymphocyte separation medium (DAKEWE). DCs were isolated from the lung mononuclear cells using CD11c magnetic beads (Miltenyi Biotec). The cells from each group of mice were mixed and analyzed. Flow cytometry analyses were performed to identify the purity of the dendritic cells. CD11c-PE (eBioscience) was used to label the DCs. CD80-FITC, CD86-PE, and CD83-FITC (eBioscience) were used to measure DC maturation.

### Statistical analysis

Statistical analysis was performed using SPSS 17.0 software. The differences between the three or four groups were determined via one-way ANOVA. A P value<0.05 was considered statistically significant. “*” denotes P < 0.05 and “**” denotes P < 0.01. The presented data are representative of at least three separate and repeated experiments.

## Results

### AhR activation by TCDD promotes the expression of CYP1a1 and CYP1b1 genes

Activated AhR binds to its dimerization partner, which enhances the expression of the target genes, such as cytochrome P450 1a1 (CYP1a1) and CYP1b1 [12]. To determine whether AhR was activated by TCDD, we first assessed the CYP1a1 and CYP1b1 gene expression. As shown in Fig 1B–1D, the expressions of CYP1a1 and CYP1b1 genes were dramatically increased in the spleens, lungs and lung-draining mediastinal lymph nodes from the TCDD-treatment mice compared with the non-treatment mice. These findings indicate that AhR was clearly activated by TCDD treatment.

### TCDD-induced AhR activation alleviates non-eosinophilic airway inflammation and airway hyperresponsiveness

**Fig 2:**
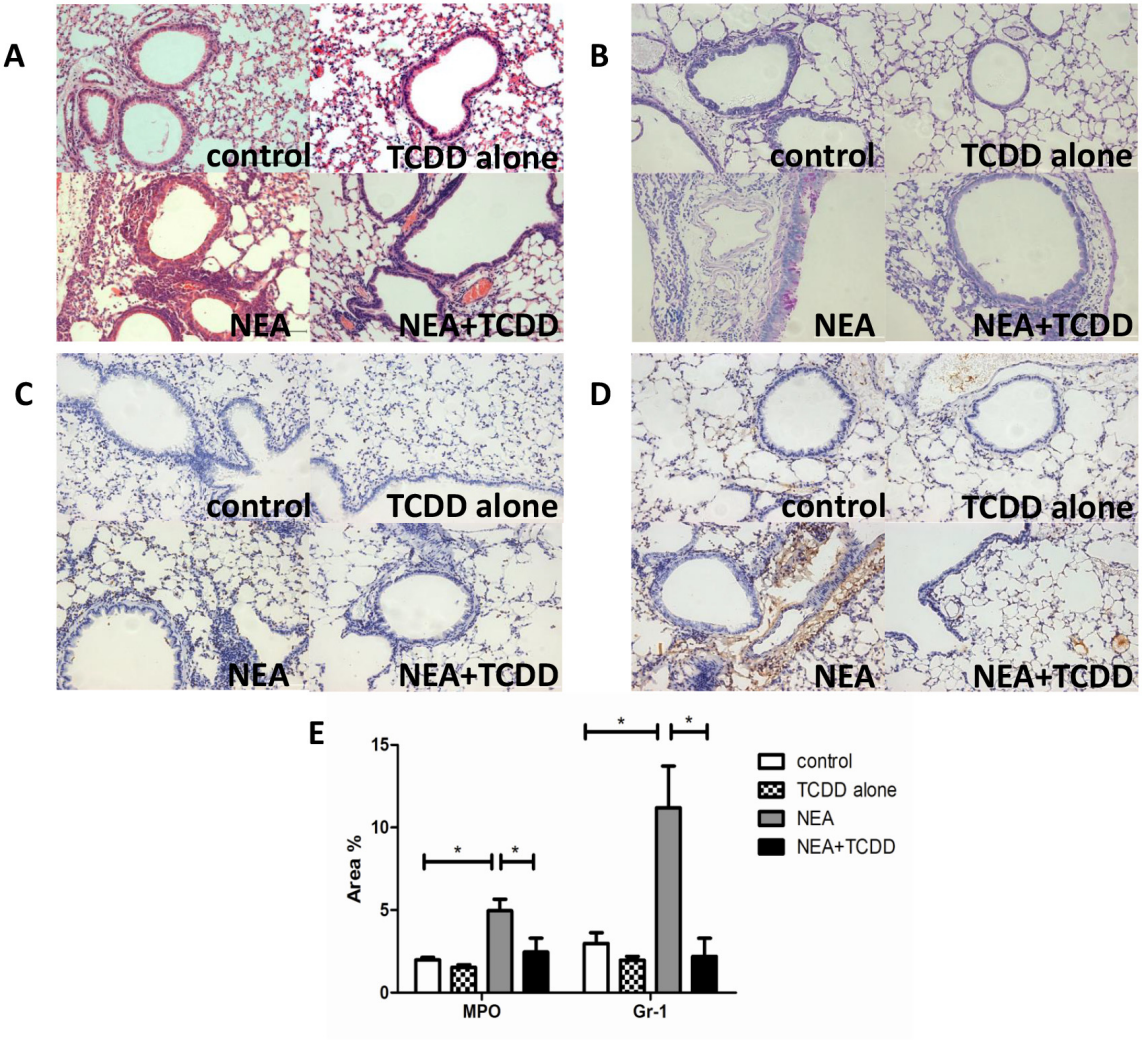
TCDD-induced AhR activation reduced non-eosinophilic airway inflammation. (A) Lung histology was analyzed via HE staining. (B) Lung histology was analyzed via PAS staining. (C) Lung histology was analyzed via neutrophilic marker-MPO staining. (D) Lung histology was analyzed via neutrophilic marker-Gr-1 staining. (E) Quantitative analysis of neutrophil infiltration in the lungs. The percentage of the positive staining area of the airway was counted from 10 randomly selected fields per section. *P<0.05.

**Fig 3:**
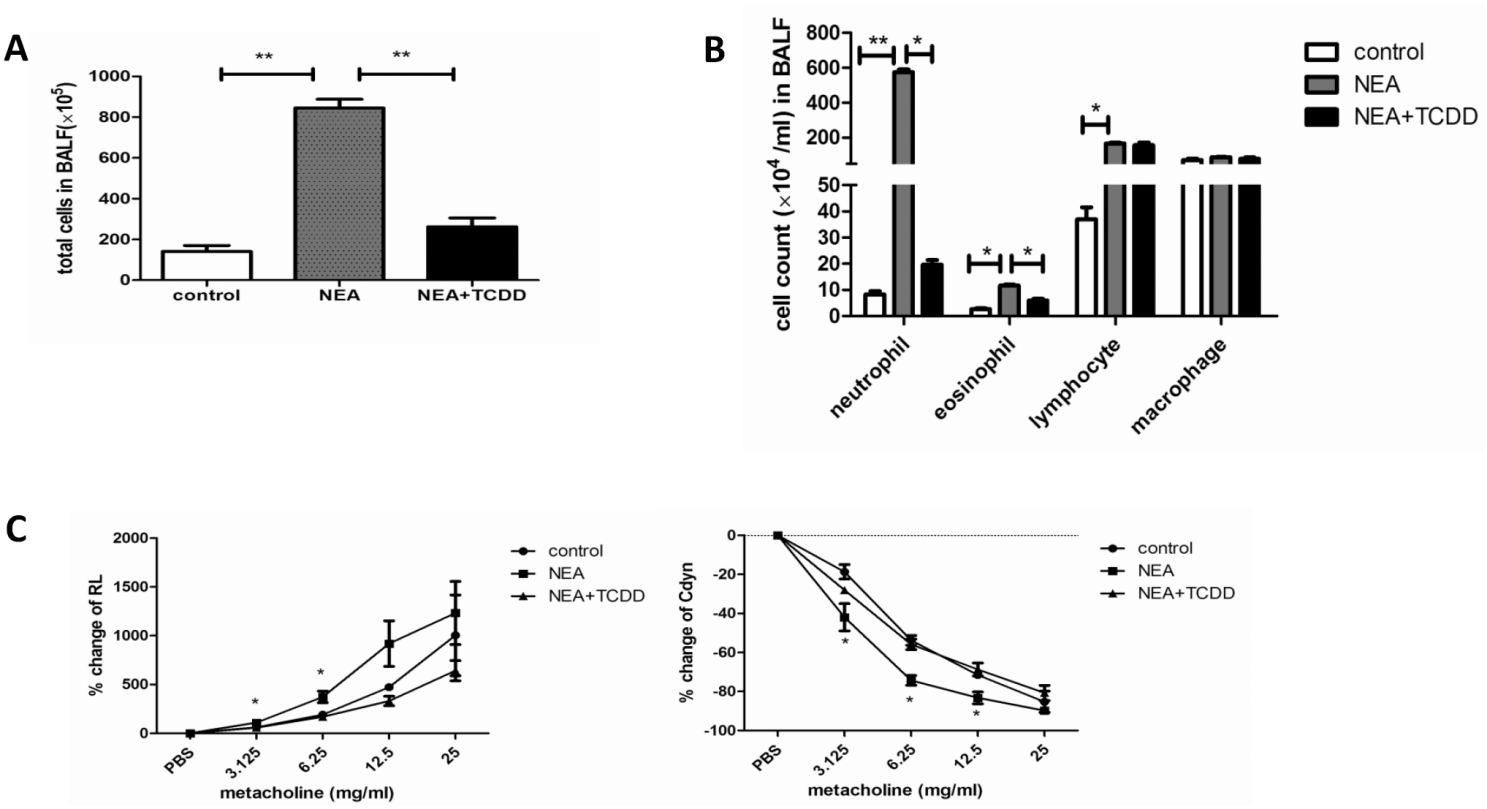
TCDD-induced AhR activation reduced non-eosinophilic airway inflammation and airway hyperresponsiveness. (A-B) Total cells and cellular composition in BALF were assessed using cytospin with May-Gruenwald Giemsa staining. (C) Airway responsiveness to aerosolized methacholine was evaluated using a Buxco’s modular and invasive system. Changes in RL and Changes in Cdyn. N = 5 per group and *P<0.05, **P<0.01.

The AhR is expressed in lung tissue [21, 22], and AhR activation plays a key role in the inflammatory reaction [17]. Thus, we investigated the regulatory function of TCDD in non-eosinophilic airway inflammation. Six-wk-old mice were intranasally sensitized with OVA and LPS, and TCDD was gavaged one day prior to being sensitized and challenged. The lung histology was analyzed via HE or PAS staining. As shown in Fig 2A and 2B, the inflammatory cells and mucus production were not significantly different in the lungs between the control and TCDD alone groups. However, these variables were substantially decreased in the lungs from the TCDD-treatment OVA/LPS sensitized mice compared with the non-treatment mice. Furthermore, we analyzed the neutrophil infiltration in the lungs by MPO and Gr-1 staining. As shown in Fig 2C and 2D, the neutrophil infiltration was also significantly decreased in the lungs from the TCDD-treatment OVA/LPS sensitized mice compared with the non-treatment mice. A quantitative analysis indicated the same results (Fig 2E). Through an analysis of the cellular composition in BALF, we determined that eosinophils and neutrophils were both significantly decreased in the TCDD-treatment group compared with the non-treatment group (Fig 3A and 3B). When challenged with various concentrations of methacholine, the RL was significantly decreased, whereas the Cdyn was significantly increased in the TCDD-treatment group compared with the non-treatment group (Fig 3C). Taken together, these findings suggest that TCDD-induced AhR activation prevents the development of allergen-induced non-eosinophilic airway inflammation and airway hyperresponsiveness.

### TCDD-induced AhR activation inhibits Th17 cytokine expression

**Fig 4:**
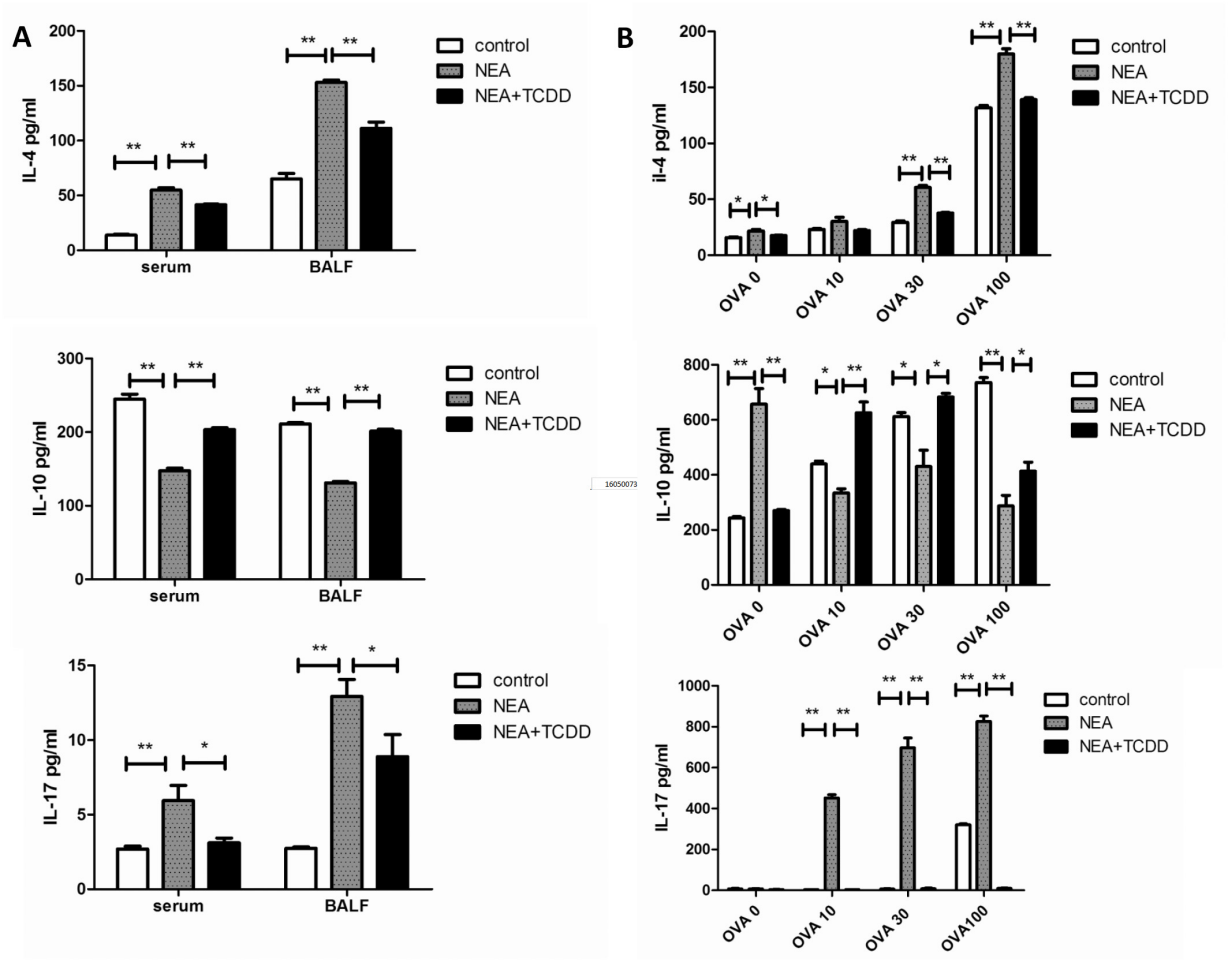
TCDD-induced AhR activation enhanced IL-10 expression and inhibited IL-17 and IL-4 expression. (A) Cytokine expression in the serum and BALF was determined via ELISA. (B) Cytokine expression from the OVA-specific T cells was determined via ELISA. N = 5 per group and *P<0.05, **P<0.01.

Cytokines play an important role in the modulation of the immune response, and Th17 has been demonstrated to evoke neutrophil recruitment [23]. Thus, we subsequently investigated whether TCDD-induced AhR activation has an impact on Th17 cytokine expression in non-eosinophilic airway inflammation. As shown in Fig 4A, the expression of IL-17 was significantly reduced in the serum and BALF from the TCDD-treatment group compared with the non-treatment group. Moreover, the production of IL-4, a Th2 cytokine, was significantly decreased compared with the non-treatment group. However, IL-10, an important pleiotropic immunoregulatory cytokine, which inhibits pro-inflammatory cytokine synthesis [24], was significantly increased compared with the non-treatment group. Consistently, following ex vivo OVA restimulation, the splenocytes and lung-draining mediastinal lymph node cells from the TCDD-treatment mice also exhibited substantially decreased IL-17 and IL-4 production and significantly increased IL-10 production (Fig 4B). Collectively, these findings indicate that TCDD-induced AhR activation regulates non-eosinophilic airway inflammation, potentially through the inhibition of pro-inflammatory cytokine synthesis.

### TCDD-induced AhR activation regulates Th17 and Treg differentiation

**Fig 5:**
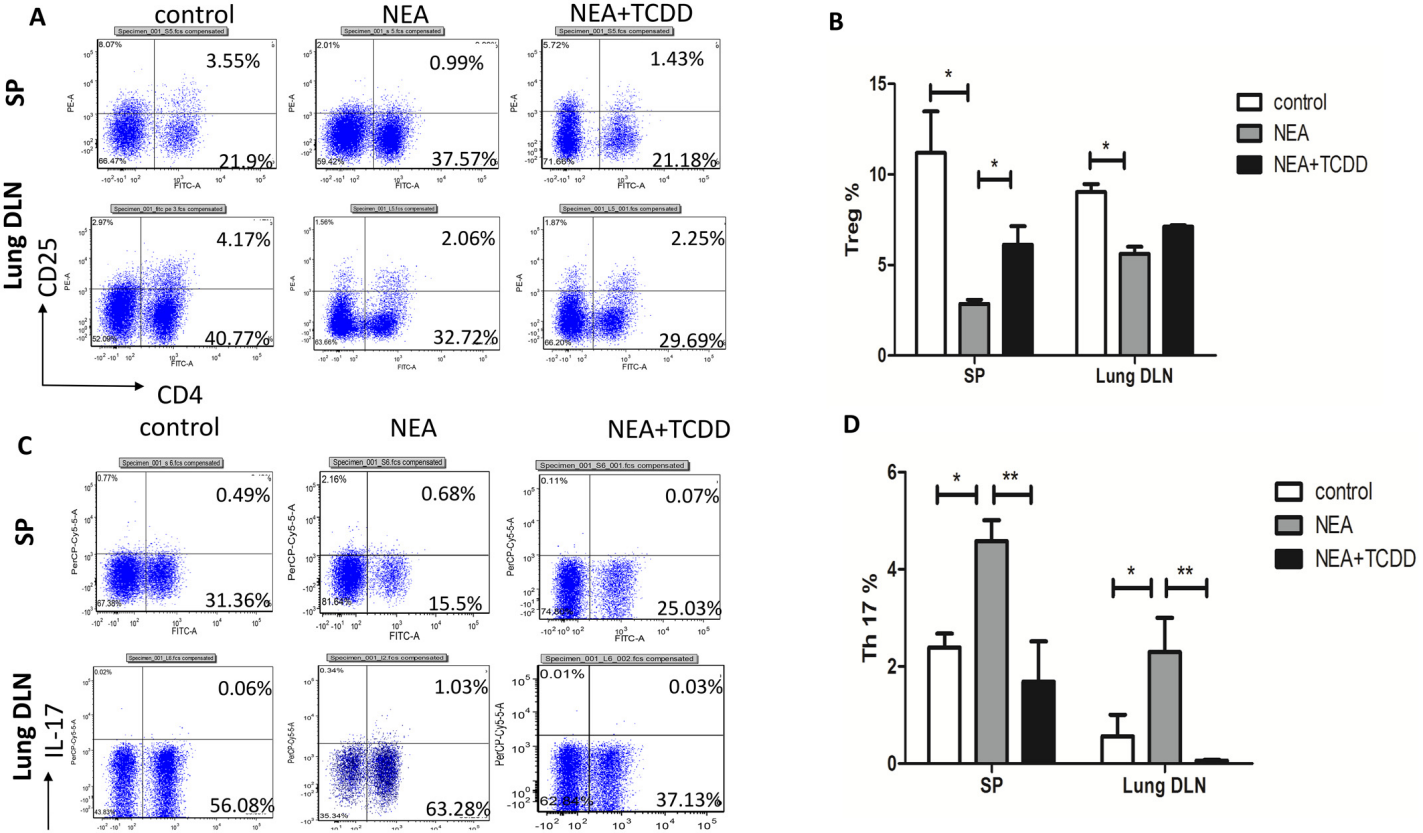
TCDD-induced AhR activation promoted Treg differentiation and inhibited Th17 differentiation. Cells from the spleens and lung-draining mediastinal lymph nodes were restimulated with leukocyte activation cocktail for 5 hours. (A-B) Five hours later, CD4+CD25+ cells were analyzed via cell surface staining. (C-D) IL-17-producing cells were analyzed via intracellular staining. The numbers within the quadrants indicate the percentage of positive cells in the total cells; the percentage of positive cells in the CD4+ cells (Treg% and Th17%) was subsequently calculated. N = 5 per group and *P<0.05, **P<0.01.

**Fig 6:**
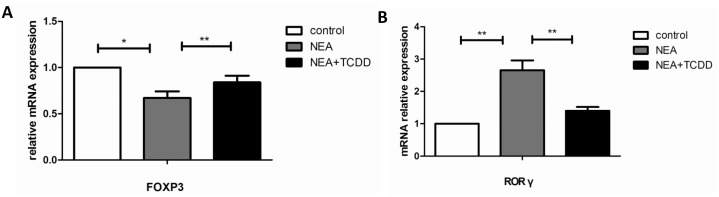
TCDD-induced AhR activation promoted FOXP3 expression and inhibited RORγ expression. Cells from the spleens were restimulated with leukocyte activation cocktail for 5 hours. (A) mRNA expression of FOXP3 in lymphocytes was determined via real-time PCR. (B) mRNA expression of RORγ in lymphocytes was determined via real-time PCR. N = 5 per group and *P<0.05, **P<0.01.

AhR activation plays an important role in the regulation of the immune system [13, 14]; thus, we subsequently investigated whether TCDD-induced AhR activation was involved in T cell differentiation in non-eosinophilic airway inflammation. Cells from the spleens and lung-draining mediastinal lymph nodes were restimulated with leukocyte activation cocktail 2 μl/ml for 5 hours, and the intracellular cytokine expression patterns were assessed. The lymphocytes from the OVA/LPS sensitized mice exhibited induced IL-17-producing cells and reduced CD4+CD25+Tregs. Following TCDD treatment, the IL-17-producing cells were substantially decreased and the CD4+CD25+Treg cells were significantly increased (Fig 5A–5D), which is consistent with a previous report by Singh *et al* [25]. Furthermore, we assessed the gene expression in the lymphocytes from the spleens that were restimulated with leukocyte activation cocktail for 5 hours. The RORγ mRNA expression was significantly decreased and FOXP3 was significantly increased in the lymphocytes from the mice treated with TCDD compared with the non-treatment mice (Fig 6A and 6B). Thus, TCDD-induced AhR activation may inhibit Th17 differentiation but enhance Treg differentiation.

### TCDD-induced AhR activation may not inhibit dendritic cell maturation

**Fig 7:**
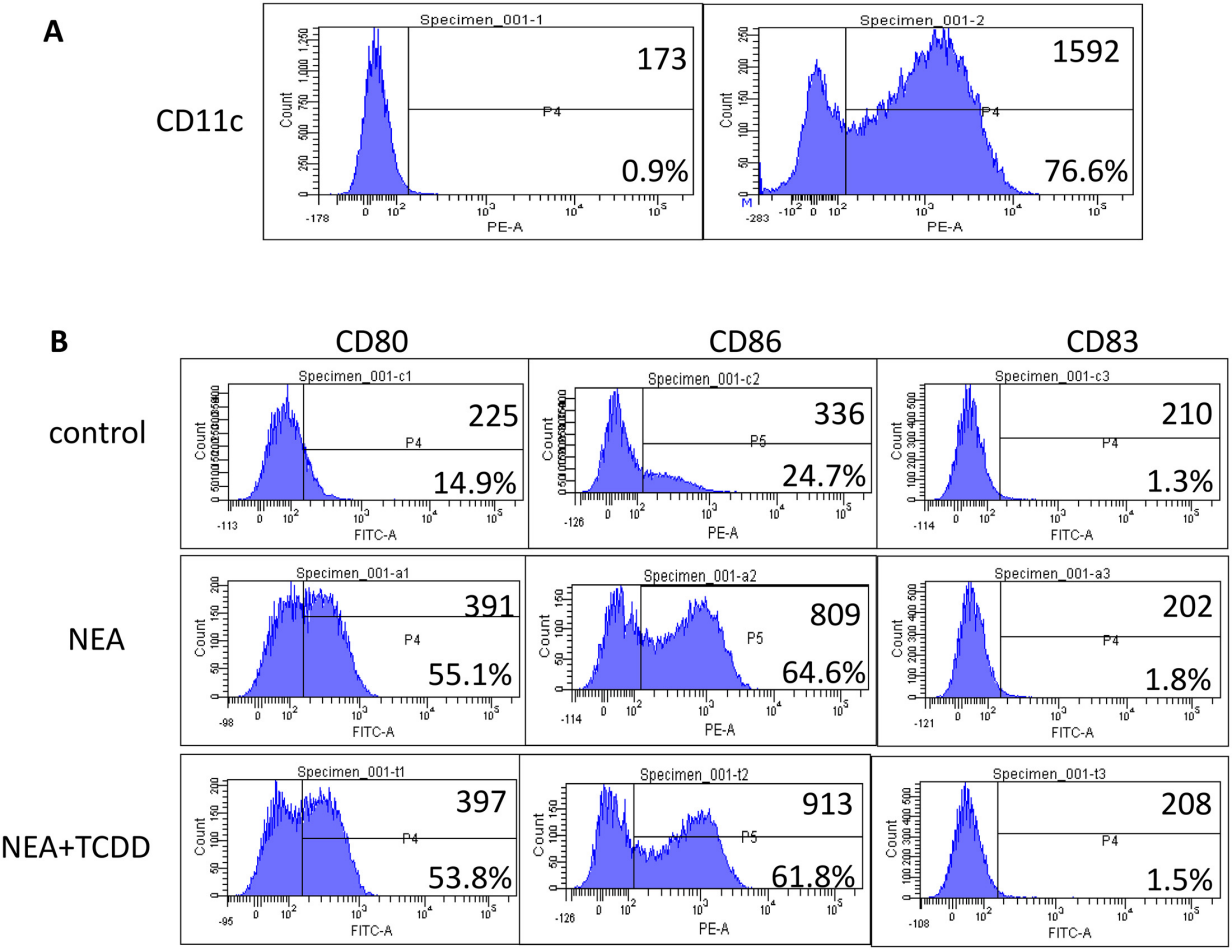
Effect of TCDD-induced AhR activation on the maturation of dendritic cells. Lung mononuclear cells were harvested using lymphocyte separation medium. DCs were isolated from lung mononuclear cells using CD11c magnetic beads (Miltenyi Biotec). (A) Flow cytometry analyses were performed to determine the purity of the dendritic cells. (B) Frequencies of CD80+, CD86+, and CD83+ cells on dendritic cells were determined via flow cytometry.

Dendritic cells (DCs) play an important role in the induction of tolerance or allergic sensitization to allergens [6]; thus, we investigated the maturation of dendritic cells in non-eosinophilic airway inflammation. DCs were isolated from lung mononuclear cells using magnetic activated cell sorting (MACS). Flow cytometry analyses confirmed that the proportion of CD11c+ cells was greater than 70% (Fig 7A). Furthermore, the flow cytometry analysis indicated that the expression CD80, CD86 and CD83 on dendritic cells was significantly induced in the OVA/LPS sensitized mice. However, the expression levels were not substantially different following TCDD treatment (Fig 7B). Therefore, dendritic cell maturation may not be inhibited by AhR activation.

## Discussion

Recently, AhR activation has been demonstrated to regulate T cell differentiation [13, 14]. In the present study, we demonstrate that TCDD-induced AhR activation reduced non-eosinophilic airway inflammation via the inhibition of Th17 differentiation and IL-17 expression and the promotion of Treg differentiation and IL-10 production. Furthermore, our findings indicate that AhR activation may not inhibit dendritic cell maturation in a non-eosinophilic asthma model.

Non-eosinophilic asthma, which is mainly characterized by neutrophilic airway inflammation, is an important inflammatory subtype of asthma. It is associated with steroid-resistance [26]. Following an antigen challenge, a substantial number of mucus production and inflammatory cells, including both eosinophils and neutrophils, infiltrated into the lung tissue in the OVA/LPS sensitized mice. However, TCDD administration reversed allergen-induced non-eosinophilic airway inflammation. In addition, the airway hyperresponsiveness was substantially decreased by treatment with TCDD. These findings collectively suggest that TCDD-induced AhR activation alleviates the severity of non-eosinophilic asthma.

Allergic asthma has traditionally been associated with the imbalance of Th1 and Th2 cell differentiation, which results in eosinophilic airway inflammation. However, recent experimental and clinical evidence indicates that severe asthma is more prone to neutrophilic airway inflammation, which is related to Th17 cell responses [23]. Eosinophilic airway inflammation, which is driven by type-2 immune responses, is characterized by the production of IL-4, IL-5, and IL-13, whereas neutrophilic airway inflammation, which is driven by type-17 immune responses, is characterized by the production of IL-17. Consistent with the cellular profiles in BALF, the production of the Th2 and Th17 cytokines was substantially decreased in the serum and BALF from the TCDD-treatment mice. The same results were demonstrated via the ex vivo OVA restimulation of lung-draining mediastinal lymph node cells and splenocytes. Thus, TCDD-induced AhR activation alleviates non-eosinophilic airway inflammation potentially via the inhibition of both Th2-type and Th17-type immune responses. Furthermore, using flow cytometry, we demonstrated that IL-17-producing cells were substantially decreased and CD4+CD25+Tregs were significantly increased following TCDD treatment. Moreover, we have tentatively identified these cells as Tregs in the absence of FoxP3 staining. The RT-PCR results indicated that the mRNA expression of RORγ was significantly decreased and FOXP3 was significantly increased in the lymphocytes restimulated via a leukocyte activation cocktail from the mice treated with TCDD compared with the non-treatment mice. Therefore, TCDD-induced AhR activation substantially affects the immunoregulatory function of non-eosinophilic asthma. These findings are consistent with a recent report that TCDD ameliorated colitis via the up-regulation of Tregs and down-regulation of Th17 cells [25].

Dendritic cells (DCs) are important for the initiation of T cell-dependent immunity, and AhR activation has been demonstrated to affect DC functions [27]. Finally, we investigated the effect of TCDD-induced AhR activation on DC maturation in non-eosinophilic airway inflammation. DCs were isolated from lung mononuclear cells using magnetic activated cell sorting (MACS). However, the expressions of CD80, CD86 and CD83 on dendritic cells were not significantly changed by TCDD-induced AhR activation, which suggests that DC maturation may not be inhibited by AhR activation. Previous studies have demonstrated that the chemotactic migration of DCs also plays an important role in the immune pathogenesis of asthma [28]; thus, whether AhR activation disturbs the chemotactic migration of DCs requires further investigation.

In conclusion, our study demonstrates that TCDD-induced AhR activation inhibits the development of non-eosinophilic airway inflammation by regulating the differentiation of Th17 and Tregs. These findings may provide a new therapeutic approach for non-eosinophilic asthma.

## References

[1] Anderson GP. Endotyping asthma: new insights into key pathogenic mechanisms in a complex, heterogeneous disease. Lancet.2008; 372:1107–1119. 10.1016/S0140-6736(08)61452-X 18805339

[2] Holgate ST, Arshad HS, Roberts GC, Howarth PH, Thurner P, Davies DE. A new look at the pathogenesis of asthma. Clin Sci (Lond).2010; 118:439–450. 10.1042/CS20090474 PMC2805922 20025610

[3] McGrath KW, Icitovic N, Boushey HA, Lazarus SC, Sutherland ER, Chinchilli VM, et al A large subgroup of mild-to-moderate asthma is persistently noneosinophilic. Am J Respir Crit Care Med.2012; 185:612–619. 10.1164/rccm.201109-1640OC 22268133 PMC3326288

[4] Aujla SJ, Alcorn JF. T(H)17 cells in asthma and inflammation. Biochim Biophys Acta. 2011; 1810:1066–1079. 10.1016/j.bbagen.2011.02.002 21315804

[5] Trejo Bittar HE, Yousem SA, Wenzel SE. Pathobiology of severe asthma. Annu Rev Pathol.2015; 10:511–545. 10.1146/annurev-pathol-012414-040343 25423350

[6] Vroman H, van den Blink B, Kool M. Mode of dendritic cell activation: the decisive hand in Th2/Th17 cell differentiation. Implications in asthma severity? Immunobiology.2015; 220:254–261. 10.1016/j.imbio.2014.09.016 25245013

[7] Matsumoto K, Inoue H, Fukuyama S, Kan-O K, Eguchi-Tsuda M, Matsumoto T, et al Frequency of Foxp3+CD4+CD25+ T cells is associated with the phenotypes of allergic asthma. Respirology.2009; 14:187–194. 10.1111/j.1440-1843.2008.01472.x 19192224

[8] Palomares O, Martín-Fontecha M, Lauener R, Traidl-Hoffmann C, Cavkaytar O, Akdis M, et al Regulatory T cells and immune regulation of allergic diseases: roles of IL-10 and TGF-*β*. Genes Immun.2014; 15:511–520. 10.1038/gene.2014.45 25056447

[9] Tian Y. Ah receptor and NF-kappaB interplay on the stage of epigenome. Biochem Pharmacol.2009; 77: 670–680. 10.1016/j.bcp.2008.10.023 19014911

[10] Hahn ME. Aryl hydrocarbon receptors: diversity and evolution. Chem Biol Interact.2002; 141: 131–160. 12213389 10.1016/s0009-2797(02)00070-4

[11] Chiba T, Uchi H, Tsuji G, Gondo H, Moroi Y, Furue M. Arylhydrocarbon receptor (AhR) activation in airway epithelial cells induces MUC5AC via reactive oxygen species (ROS) production. Pul Pharmacol Ther.2011; 24:133–140. 10.1016/j.pupt.2010.08.002 20709182

[12] Mimura J, Fujii-Kuriyama Y. Functional role of AhR in the expression of toxic effects by TCDD. Biochim Biophys Acta. 2003; 1619:263–268. 12573486 10.1016/s0304-4165(02)00485-3

[13] Esser C, Rannug A, Stockinger B. The aryl hydrocarbon receptor in immunity. Trends Immunol.2009; 30: 447–454. 10.1016/j.it.2009.06.005 19699679

[14] Stevens EA, Mezrich JD, Bradfield CA. The aryl hydrocarbon receptor: a perspective on potential roles in the immune system. Immunology.2009; 127: 299–311. 10.1111/j.1365-2567.2009.03054.x 19538249 PMC2712099

[15] Veldhoen M, Hirota K, Westendorf AM, Buer J, Dumoutier L, Renauld JC, et al The aryl hydrocarbon receptor links TH17-cell-mediated autoimmunity to environmental toxins. Nature.2008; 453: 106–109. 10.1038/nature06881 18362914

[16] Veldhoen M, Hirota K, Christensen J, O’Garra A, Stockinger B. Natural agonists for aryl hydrocarbon receptor in culture medium are essential for optimal differentiation of Th17 T cells. J Exp Med.2009; 206: 43–49. 10.1084/jem.20081438 19114668 PMC2626686

[17] Quintana FJ, Basso AS, Iglesias AH, Korn T, Farez MF, Bettelli E, et al Control of T(reg) and T(H)17 cell differentiation by the aryl hydrocarbon receptor. Nature.2008; 453:65–71. 10.1038/nature06880 18362915

[18] Kim YK, Oh SY, Jeon SG, Park HW, Lee SY, Chun EY, et al Airway exposure levels of lipopolysaccharide determine type 1 versus type 2 experimental asthma. J Immunol. 2007; 178: 5375–5382. 17404323 10.4049/jimmunol.178.8.5375

[19] Pichavant M, Goya S, Hamelmann E, Gelfand EW, Umetsu DT. Animal models of airway sensitization. Curr Protoc Immunol.2007; Chapter 15:Unit 15 18. 10.1002/0471142735.im1518s79 18432985

[20] Glaab T, Daser A, Braun A, Neuhaus-Steinmetz U, Fabel H, Alarie Y, et al Tidal midexpiratory flow as a measure of airway hyperresponsiveness in allergic mice. Am J Physiol Lung Cell Mol Physiol.2001;280:L565–573. 11159041 10.1152/ajplung.2001.280.3.L565

[21] Dolwick KM, Schmidt JV, Carver LA, Swanson HI, Bradfield CA. Cloning and expression of a human Ah receptor cDNA. Mol Pharmacol.1993;44:911–917. 8246913

[22] Li W, Donat S, Dohr O, Unfried K, Abel J. Ah receptor in different tissues of C57BL/6J and DBA/2J mice: use of competitive polymerase chain reaction to measure Ah-receptor mRNA expression. Arch Biochem Biophys.1994; 315:279–284. 7986069 10.1006/abbi.1994.1501

[23] McKinley L, Alcorn JF, Peterson A, Dupont RB, Kapadia S, Logar A, et al TH17 cells mediate steroid-resistant airway inflammation and airway hyperresponsiveness in mice. J Immunol.2008; 181: 4089–4097. 18768865 10.4049/jimmunol.181.6.4089 PMC3638757

[24] Borish L, Aarons A, Rumbyrt J, Cvietusa P, Negri J, Wenzel S. Interleukin-10 regulation in normal subjects and patients with asthma. J Allergy Clin Immunol. 1996;97:1288–1296. 8648025 10.1016/s0091-6749(96)70197-5

[25] Singh NP, Singh UP, Singh B, Price RL, Nagarkatti M, Nagarkatti PS. Activation of aryl hydrocarbon receptor (AhR) leads to reciprocal epigenetic regulation of FoxP3 and IL-17expression and amelioration of experimental colitis. PLoS One. 2011; 6:e23522 10.1371/journal.pone.0023522 21858153 PMC3156147

[26] McGrath KW, Icitovic N, Boushey HA, Lazarus SC, Sutherland ER, Chinchilli VM, et al A large subgroup of mild-to-moderate asthma is persistently noneosinophilic. Am J Respir Crit Care Med.2012;185:612–619. 10.1164/rccm.201109-1640OC 22268133 PMC3326288

[27] Schulz VJ, van Roest M, Bol-Schoenmakers M, van Duursen MB, van den Berg M, Pieters RH, et al Aryl hydrocarbon receptor activation affects the dendritic cell phenotype and function during allergic sensitization. Immunobiology.2013; 218:1055–1062. 10.1016/j.imbio.2013.01.004 23433705

[28] Pichavant M, Charbonnier AS, Taront S, Brichet A, Wallaert B, Pestel J, et al Asthmatic bronchial epithelium activated by the proteolytic allergen Der p 1 increases selective dendritic cell recruitment. J Allergy Clin Immunol. 2005;115:771–778. 15805997 10.1016/j.jaci.2004.11.043

